# Harnessing III-Nitride Built-In Field in Multi-Quantum Well LEDs

**DOI:** 10.1021/acsami.4c02084

**Published:** 2024-04-26

**Authors:** Mikołaj Chlipała, Henryk Turski

**Affiliations:** † Institute of High Pressure Physics, Polish Academy of Sciences, Sokołowska 29/37, 01-142 Warsaw, Poland; ‡ Department of Electrical and Computer Engineering, Cornell University Ithaca, New York 14853, United States

**Keywords:** GaN, LED, PA-MBE, multiple QW, nitride semiconductors, InGaN quantum wells, epitaxy

## Abstract

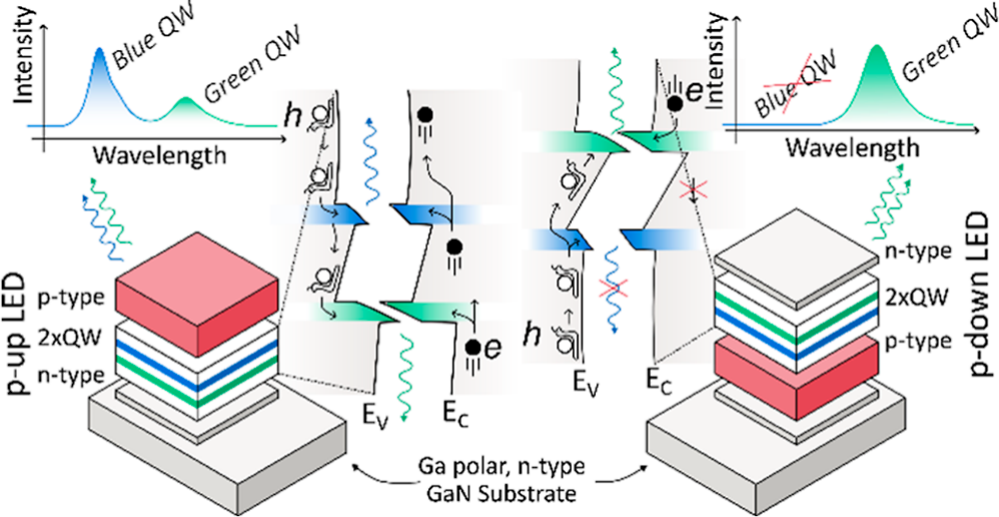

III-nitrides possess several unique qualities, which allow them to make the world brighter, but their uniqueness is not always beneficial. The uniaxial nature of the wurtzite crystal leads to strikingly large electric polarization fields, which along with the high acceptor ionization energy cause low injection efficiency and uneven carrier distribution for multiple quantum well (QW) light emitting devices. In this work, we explore the carrier distribution in Ga-polar LED in two configurations: standard “p-up” and “p-down”, which is accomplished by utilizing a bottom-tunnel junction. This enables the inversion of the sequence of the p and n layers while altering the direction of the current flow with respect to the inherent polarization. To probe the carrier distribution two, color-coded QWs are used in alternating sequences. Our study reveals that for “p-down” devices carrier transport through multiple QWs is limited by the potential barrier at the QW interface, which is in contrast to results for “p-up” structures, where hole mobility is the bottleneck. Moreover, investigated “p-down” LEDs exhibit an extremely low turn-on voltage.

## 1 Introduction

**Figure 1:**
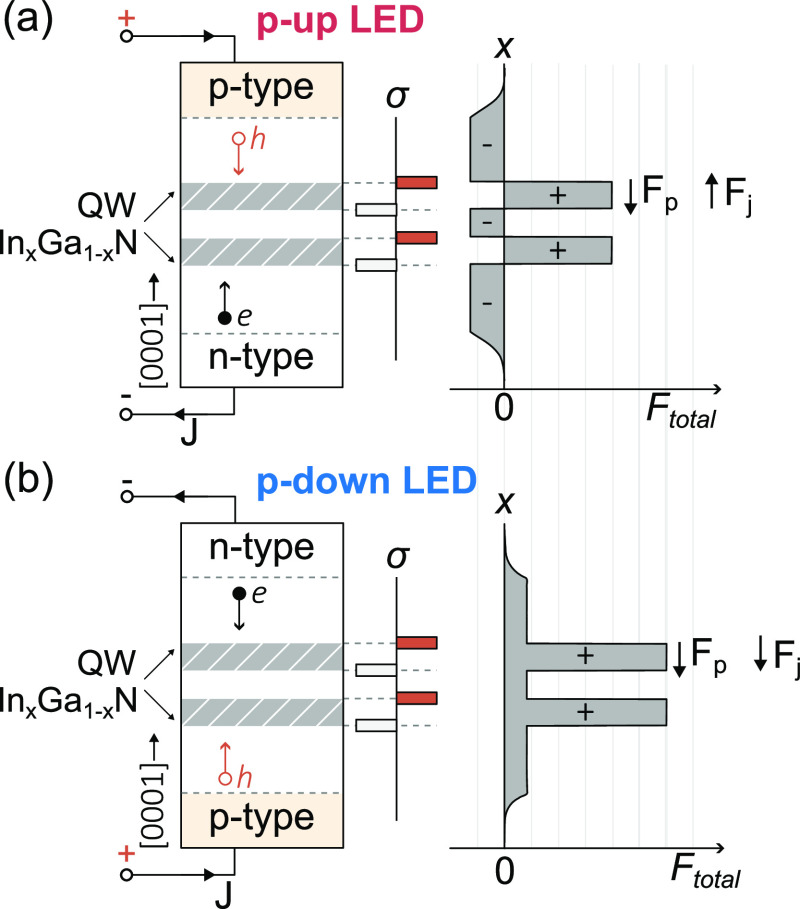
On the left, simplified structures of LED with InGaN QW and GaN barriers on the Ga polar substrate for (a) standard p-up LED, (b) p-down LED with corresponding: σ-polarization sheet charge at QW interfaces, total electric field, direction of built-in electric fields , and junction field  without an external bias.

III-nitride semiconductors exhibit a remarkable array of distinctive properties that have significantly impacted the field of optoelectronics. However, some of these unique qualities, while contributing to their success, also present challenges that require careful consideration in device design. One of such characteristics arises from the noncentrosymmetric nature of their wurtzite crystal structure, which results in the strikingly high built-in electric fields  due to the spontaneous and piezoelectric polarization. This effect, although beneficial in certain applications, can be challenging in others. In the context of standard light-emitting diodes (LEDs) grown on the Ga polar substrate with a p-type layer on the top (Figure 1a), the influence of built-in polarization is widely studied.^1−3^ It leads to detrimental effects such as separation of electron and hole wave functions in quantum wells (QWs),^4^ reduced injection efficiency with electron overflow to p-type regions,^5−7^ and asymmetric carrier distribution within multiple QWs.^8−11^ However, the built-in polarization within III-nitrides can also be harnessed as a valuable design parameter, offering opportunities for tailoring device performance and functionality, making it a double-edged sword in the world of nitride semiconductor technology.

The unconventional design of “p-down” LEDs grown on Ga-polar substrates is one example of the effective exploitation of the built-in field.^12−17^ In this configuration, the sequence of p and n type layers in the LED is inverted, as illustrated in Figure 1b, contrasting it with the standard “p-up” LED. For “p-down” LEDs, both forward bias and current direction are inverted with respect to the “p-up” device, while the energy barrier positions stay the same. The presence of the energy barriers and charge injection direction influence on performance of LEDs is described in literature, starting from the beginning of the XXI century^12,18^ to modern devices with tunnel junctions (TJ).^16,19^ The authors investigated this topic using different types of experiments such as: current–voltage, parasitic recombination outside QW,^14^ capacitance–voltage, and drift-diffusion calculations.^15^

In terms of the electric field, inverted sequence of p- and n-type layers, results in junction field () and  pointing in the same direction, leading to a heightened total electric field in QW compared to the “p-up” LED. However, an external forward bias reduces the electric field in QW, moving toward a flat-band condition, thereby enhancing wave function overlap. Furthermore, in the “p-down” LED, the negative polarization charge (σ) is positioned farther away from the n-type cladding layer, creating a barrier that prevents electron overflow. Conversely, in the “p-up” LED, the same barrier prevents carrier injection. As a result, p-down LED exhibits a lower turn on voltage, higher injection efficiency, and lower parasitic recombination in cladding layers, as it is discussed in more details in refs (5,14) Moreover, the “p-down” structure grown on Ga-polar surfaces mimics the “p-up” LED grown on N-polar substrates, inheriting all the advantageous traits of N-polar built-in field alignment without the detrimental issues associated with N-polar substrates.^20^ This provides a unique opportunity to analyze carrier distribution in a high-efficiency LED with N-polar like built-in fields.

Another unique property that distinguishes III-nitrides is the substantial disproportion in the mobility and activation energy of electrons and holes within these materials. In typical III-nitride materials like gallium nitride (GaN), the electron mobility can reach values up to 2000 cm^2^/V·s, while the hole mobility typically hovers around 10–30 cm^2^/V·s, indicating a notable preference for electron transport.^21^ This significant difference between electron and hole properties poses challenges in achieving balanced carrier transport in multiple QW LEDs. Together with built-in polarization, those effects lead to a high asymmetry in charge transport through the device.

Several studies have proposed the use of multiple QW approach for N-polar^4,6^ and “p-down”^22^ configurations, which was proven before to be effective for standard “p-up” LEDs.^23^ However, is it valid to apply the same design principles for “p-down” LEDs as for conventional LEDs? In this study, we systematically investigate the carrier distribution in Ga-polar double QW LEDs by employing both experimental and theoretical approaches. We are examining two distinct constructions: “p-up”, which represents the standard approach to LEDs and acts as the reference point, and the unconventional “p-down” construction, which differs in built-in field alignment with respect to current flow direction. The indium content in the QWs was varied, resulting in one emitting blue light and the other emitting green light. By varying the QW sequence, we create four configurations that visualize carrier distribution through emission spectra. We show that proposed structures enable the investigation of the impact of a built-in field on carrier injection by tracking the emission spectra, as it correlates with the carrier distribution between multiple QWs in the device.

## 2 Experimental Section

**Figure 2:**
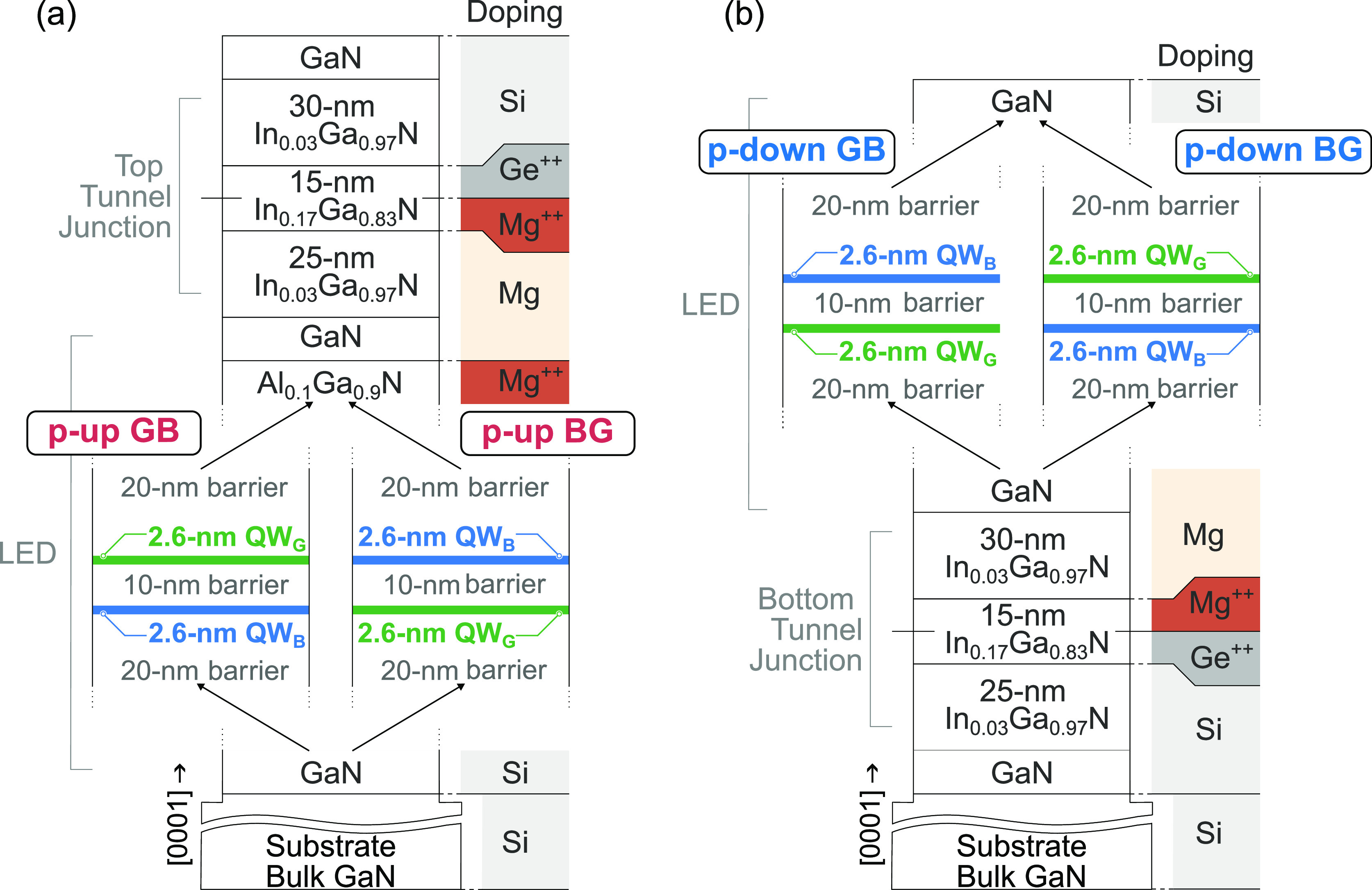
Schematic epitaxial stack for (a) “p-up” LED with the top tunnel junction and blue and green QWs (QW_B_ and QW_G_, respectively) arranged as follows: QW_G_ is closer to the p-type on the left (labeled a p-up GB), while QWB is closer to the p-type on the right (labeled a p-up BG). (b) Bottom tunnel junction p-down LED. The QWs are arranged as follows: QW_G_ is closer to the p-type (labeled p-down GB) on the left, while QW_B_ is closer to the p-type (labeled p-down BG) on the right.

Four LED structures were grown on free-standing Ga-polar GaN substrates using plasma-assisted molecular beam epitaxy (PA-MBE). This is hydrogen-free technique that enables the growth of high-quality buried p-type layers and highly doped TJ.^24,25^ Despite substantial successes reported for “p-down” LEDs grown by metal–organic vapor phase epitaxy,^22^ it is still PA-MBE that offers a more straightforward approach to grow them. Schematic structures of the investigated samples are listed in Figure 2. Each structure incorporates a double, 2.6 nm thick QW, each with different compositions. One QW is composed of In_0.17_Ga_0.83_N and emits in the blue spectral region while the other is composed of In_0.22_Ga_0.78_N layers and operates at a longer wavelength in the green spectral range. What is changed among the series is the sequence of QWs and the direction of the junction field. We denote each structure according to the sequence of the corresponding QWs with respect to the p-type layer position, where “B” is for blue QW and “G” for green and the first capital letter describes QW closer to the p-type.

Two of the samples have p-type layers above QW followed by TJ on the top. This type of sample is denoted as “p-up”. The active region order for these samples is BG and GB, as illustrated in Figure 2a on the left and right, respectively. Both “p-up” samples incorporate a dedicated electron-blocking layer (EBL) composed of 20 nm, Mg-doped Al_0.1_GaN, representing a conventional approach in GaN-based LEDs, as depicted in Figure 1a.

The two LEDs illustrated in Figure 2b incorporate a bottom TJ beneath the active region, with quantum wells arranged in the order GB and BG (as shown in Figure 2a on the left and right, respectively). These configurations represent the “p-down” structures, as depicted in Figure 1b. Unlike the “p-up” LEDs, these devices lack an AlGaN EBL. Both “p-up” and “p-down” LEDs share the same direction of the built-in polarization in the active region with respect to the substrate; however, they differ in the junction field direction and subsequent current flow direction.

**Figure 3:**
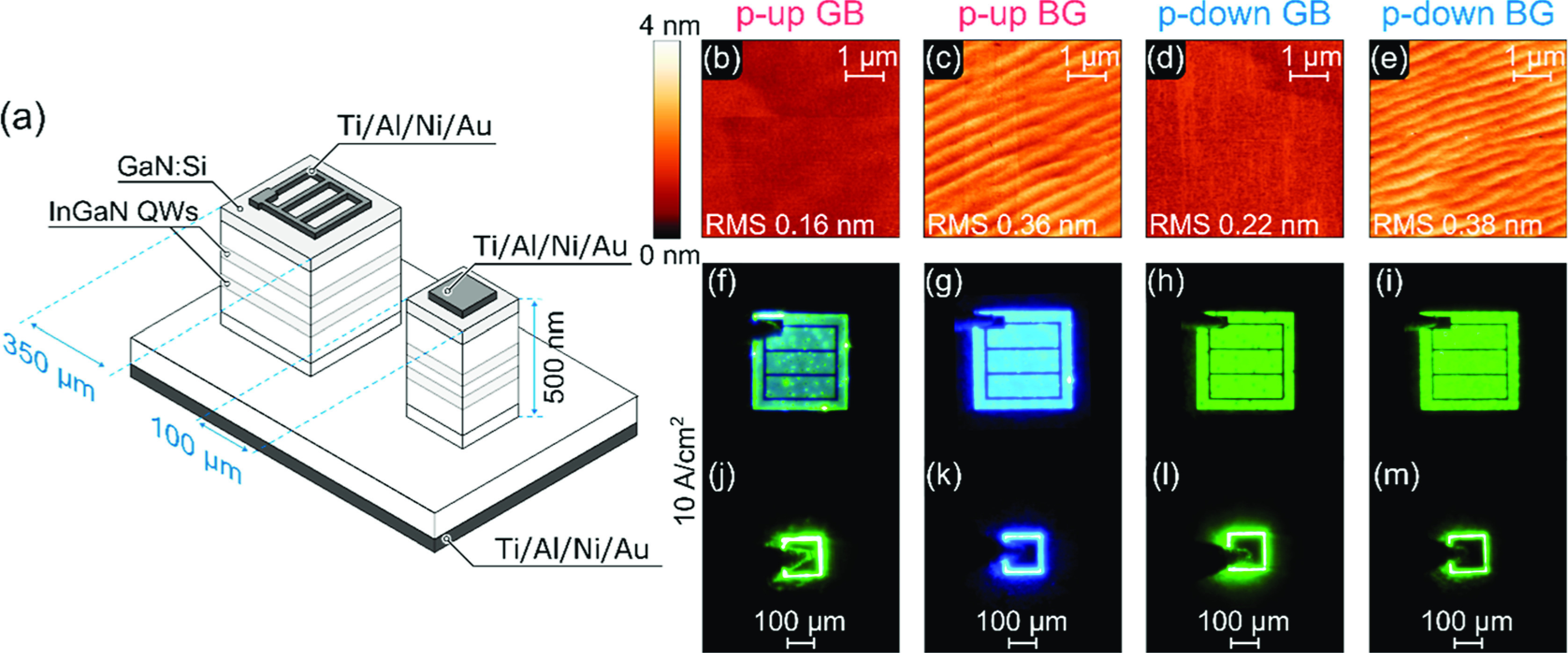
(a) Schematic structure of the processed LEDs, one 350 × 350 μm^2^ with metal grid and second, smaller 100 × 100 μm^2^ with full square metallization. (b–e) Atomic force microscopy scans of the surface morphologies and root-mean-squared (RMS) roughness. (f–i) Optical microscopy image of processed 350 × 350 μm^2^ and (j–m) 100 × 100 μm^2^ devices at 10 A/cm^2^.

Each TJ consists of a 15 nm In_0.17_Ga_0.83_N layer with Ge- and Mg-doped halves. Doping levels are 2 × 10^20^ cm^–3^ for Ge and 1 × 10^20^ cm^–3^ for Mg.^26^ It was estimated based on separate calibration samples investigated by secondary ion mass spectrometry. The TJ allows termination of all the studied LED structures with the same n-type layer that provides low resistivity and ensures uniform current spreading. For both top and bottom contacts, the same stack of Ti/Al/Ni/Au with thicknesses of 30/60/40/75 nm, respectively, was deposited. The samples were coprocessed into LEDs with mesa sizes of 100 × 100 and 350 × 350 μm^2^ both present at all wafers and featuring square metallization and grid metallization, respectively, as presented in Figure 3a.

Epitaxy was performed in a Veeco GEN20a PA-MBE system equipped with standard effusion cells and a radiofrequency nitrogen plasma source. Devices were characterized by a custom-made probe station featuring: Keithley 2410 for electrical measurements and Thorlabs S130VC optical power meter mounted in a microscope column. Spectra were collected through fiber by an Ocean Optics USB4000. The measurement setup and sample surface, which features opaque contact metal stacks on the top and bottom, were not optimized for efficient light collection and extraction. This suboptimal configuration accounts for the observed low optical power values, which are on the order of a few microwatts (μW). The optical power was scaled by the wavelength-dependent responsivity of the detector. For samples emitting both in green and blue, the contribution to total optical power of each peak was calculated based on the peak ratio obtained from spectra. The optical power was used to calculate the external quantum efficiency (EQE). The detailed description of how EQE was extracted for multicolor emission is included in the Supporting Information and in refs (27), (28). More information regarding device uniformity and statistics of measurements is included in the Supporting Information.

## 3 Results and Discussion

Post-growth, the surface quality was evaluated by atomic force microscopy (AFM). Figure 3b–e presents the surface morphologies and root-mean-square (RMS) roughness for all samples, revealing uniformly atomically flat surfaces. Real-color optical microscopy images of the processed LEDs with 350 × 350 μm^2^ and 100 × 100 μm^2^ mesas under a forward bias are depicted in Figure 3f–m. Atop the devices presented in Figure 3f–i, there is a metal grid contact deposited directly on the n-type GaN, without any additional semitransparent current spreader. The visible bright circular defects are caused by altered growth conditions beneath indium droplets that form during metal-rich PA-MBE InGaN growth. The dark circular spots are accompanied by screw and edge dislocations, which serve as nonradiative recombination centers and can contribute to the leakage current. More comprehensive descriptions of the defects observed in LEDs grown by PA-MBE can be found in ref (29). For subsequent investigations, devices with full square metal contacts (Figure 3j–m) are used as they offer more reliable and reproducible results.

**Figure 4:**
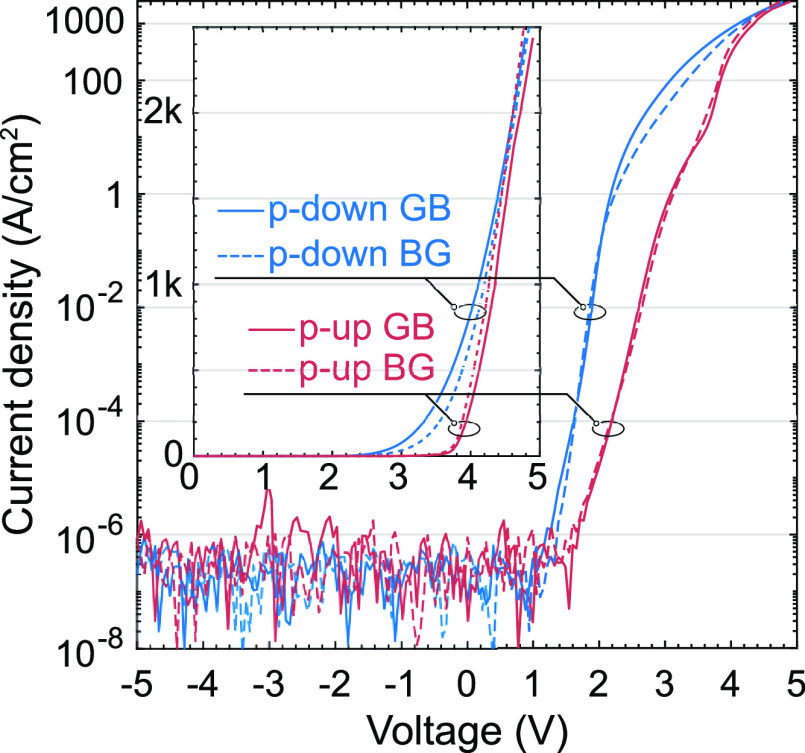
I–V characteristics in the semilogarithmic scale for 100 × 100 μm^2^ devices. The inset presents the same set of data, but on the linear scale.

Figure 4 illustrates semilogarithmic current–voltage (I–V) curves. Notably, the forward bias direction of the “p-up” LEDs is opposite to that of the “p-down” samples. To facilitate a comparison between the devices, the voltage and current signs are flipped for the “p-down” samples. All samples exhibit a low leakage current in reverse bias, reaching −5 V. A noticeable difference between the types of samples is that p-down LEDs demonstrate a lower turn-on voltage than standard p-up LEDs, attributed to the absence of usual barriers for current injection.^14^ Within a specific device type, either “p-up” or “p-down”, the order of the quantum wells (QWs) does not influence the electrical properties.

**Figure 5:**
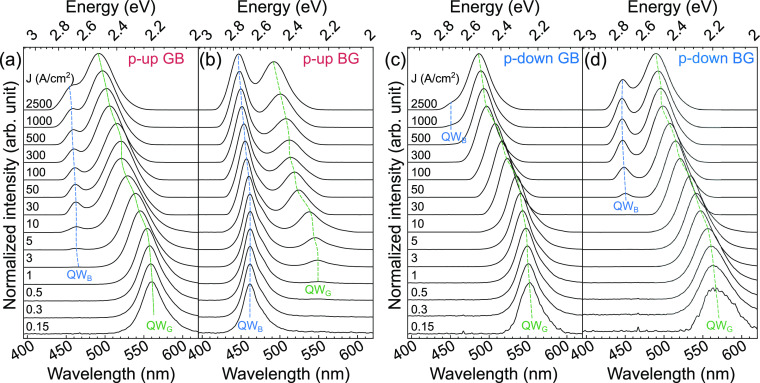
Spectra for 100 × 100 μm^2^ LEDs at current density ranging from 0.15 A/cm^2^ to 2.5 kA/cm^2^. “P-up” LED with (a) blue QW and (b) green QW closer to the p-type layers, respectively. “P-down” LED with (c) green QW and (d) blue QW closer to the p-type layers, respectively. The energy at maximum intensity is plotted in Figure 6.

Electroluminescence spectra for all LEDs at various current densities are shown in Figure 5. We started the analysis by looking at the more standard “p-up” devices. For “p-up” GB LED (Figure 5a), we can see that the peak originating from the green QW, which is closer to the p-type, dominates the spectra and that with increasing current the additional peak from the second, blue QW starts to be visible. Whereas for the sample p-up BG (Figure 5b), with a reversed order of QWs’ colors, we get reversed order of peaks in comparison to the first sample. In this case, the peak originating from the blue QW, which is closer to the p-type, dominates the spectra, and the second green peak starts to be visible at higher current densities. We observe that luminescence from the QW closest to the p-type dominates spectra for p-up samples (Figure 5a,b), regardless of the In concentration.

For emission spectra of both GB and BG “p-down” LEDs presented in Figure 5c,d, respectively, the predominant spectral contribution comes from the green quantum well (the one with the highest In content). Meanwhile, the peak originating from the blue QW becomes discernible for “p-down” BG only at a relatively high current density of 50 A/cm^2^. For the “p-down” GB device, extra blue emission does not appear up to extremely high 1 kA/cm^2^. Notably, in contrast to “p-up” LEDs, in “p-down” construction the emission peak from the QW with the highest In content dominating the spectrum, regardless of its position within the stack.

**Figure 6:**
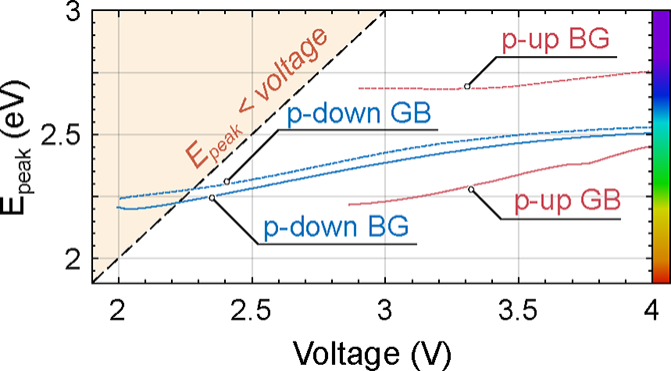
Energy at maximum electroluminescence intensity (*E*_peak_) from Figure 5 vs voltage.

Figure 6 illustrates the correlation between energy at maximum intensity (*E*_peak_) and external bias, with the highlighted area indicating where *E*_peak_ in electron volts is lower than the bias in volts. It is evident that measurable light emission occurs at lower voltages for “p-down” LEDs compared to “p-up” LEDs. Notably, both “p-down” GB and BG LEDs exhibit light emission with an energy exceeding 2.2 eV at a remarkably low voltage of 2.0 V, for current density between 0.15 and 2 A/cm^2^, whereas for “p-up” GB LED one needs 2.85 V to reach similar emission energy. This is a notable achievement, shattering the barrier for *E*_peak_ equal to the applied voltage. It is a relation regarded by many as describing an ideal device, a standard surpassed here by more than 200 meV.^19^

**Figure 7:**
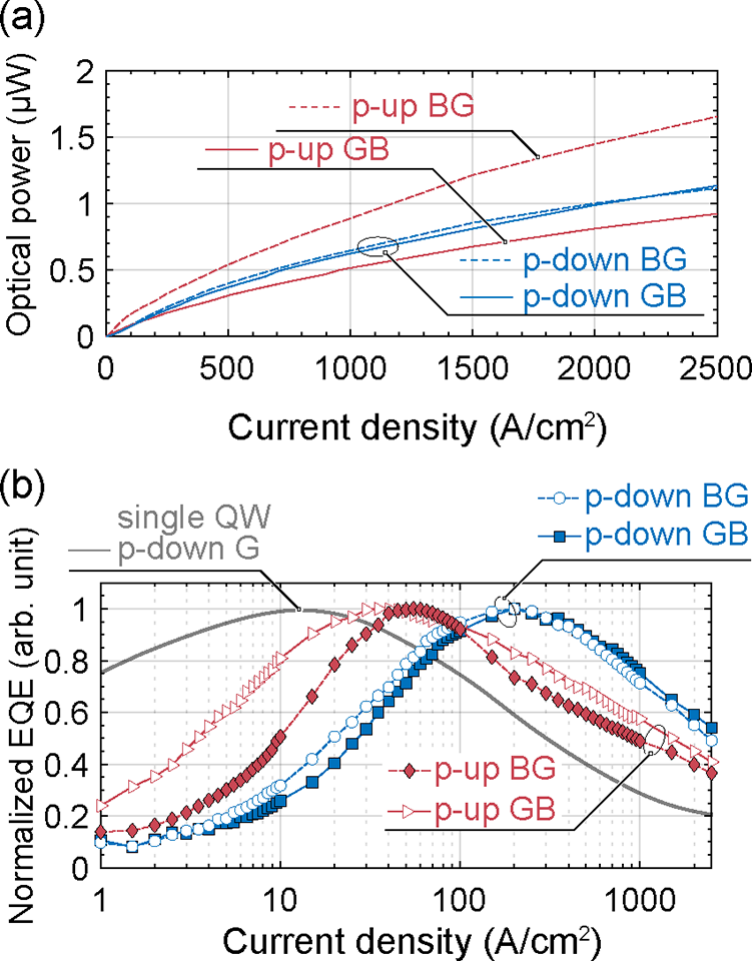
(a) Dependence of the optical power on the current density. (b) Normalized external quantum efficiency (EQE). “P-down”, single QW, and green LED was added as a reference for double QW LEDs.

The optical power versus current density is illustrated in Figure 7a. The “p-up” BG sample shows a higher light output compared to both “p-down” and “p-up” GB samples, which exhibit similar light outputs. This effect can be attributed to the dominance of the blue quantum well (QW) in the spectra for the “p-up” BG LED (Figure 5b), while in the remaining samples, the green QW dominates in the spectra, typically associated with a higher defect concentration than the blue QW with a lower In content.

The optical power was used to calulate the EQE, which is presented in Figure 7b.Supporting Information As a reference, a “p-down” LED with a single green QW (“p-down” G) is included in the EQE plot in Figure 7b. The EQE peak is observed at a current density of 12.2 A/cm2. Following the ABC model form eq 2, this suggests good QW quality despite being grown on top of a highly doped tunnel junction. For double QW LEDs, a clear trend emerges. The “p-up” devices exhibit a lower current at the EQE peak, specifically 40 and 55 A/cm^2^ for GB and BG LEDs, respectively, compared to “p-down” LEDs, where it is 200 A/cm^2^. However, this does not necessarily indicate poor QW quality in double QW LEDs. Assuming similar ABC parameters to those of the single QW LED, we can conclude that the increase in the EQE peak position can be attributed to uneven carrier concentration, with this effect being more pronounced for “p-down” LEDs.

**Figure 8:**
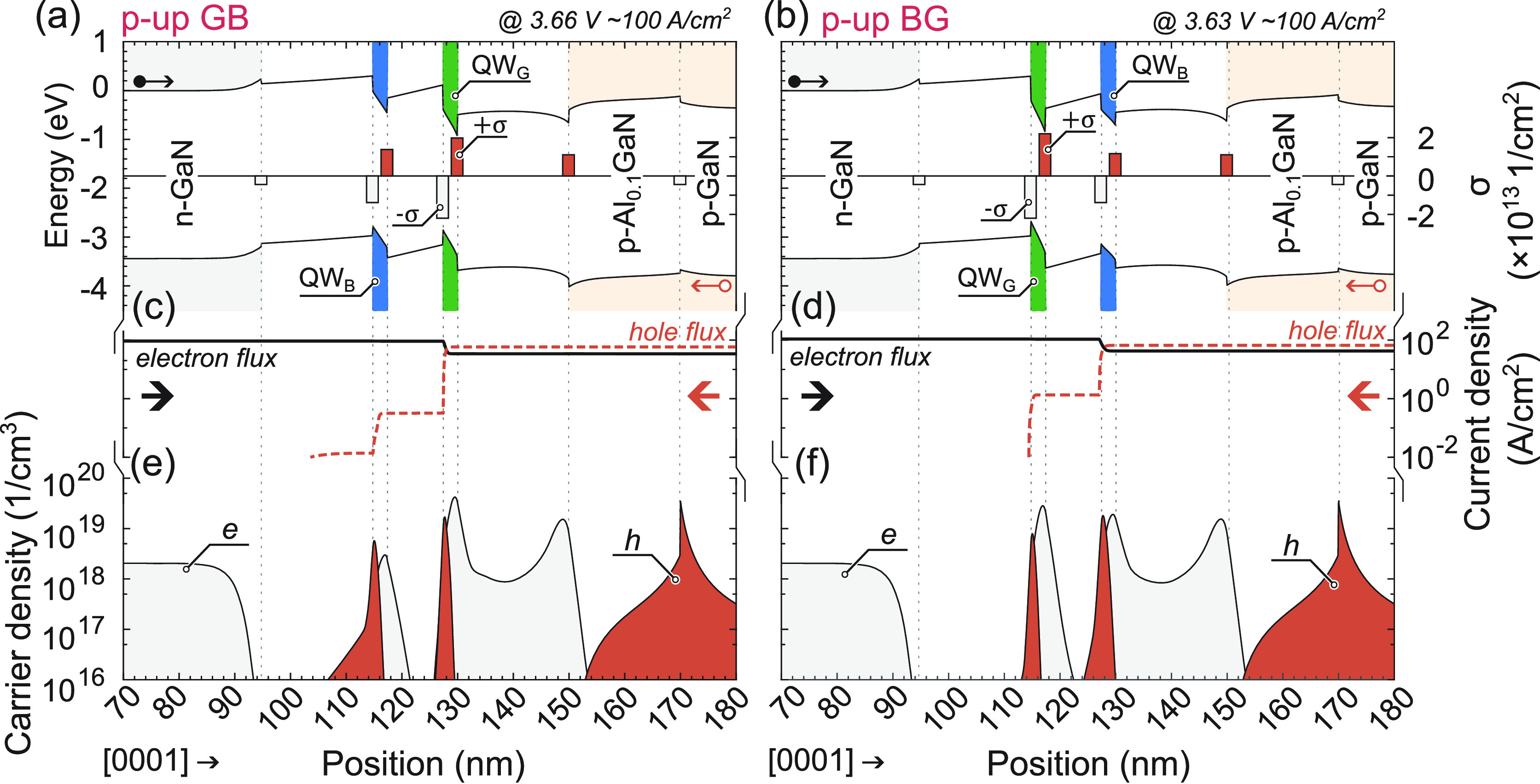
For “p-up” LEDs: (a,b) band structures at ∼100 A/cm^2^ with σ-polarization sheet charge at interfaces, (c,d) current density vs position, and (e,f) carrier density vs position. Left column describes p-up GB LED, right column describes p-up BG LED.

**Figure 9:**
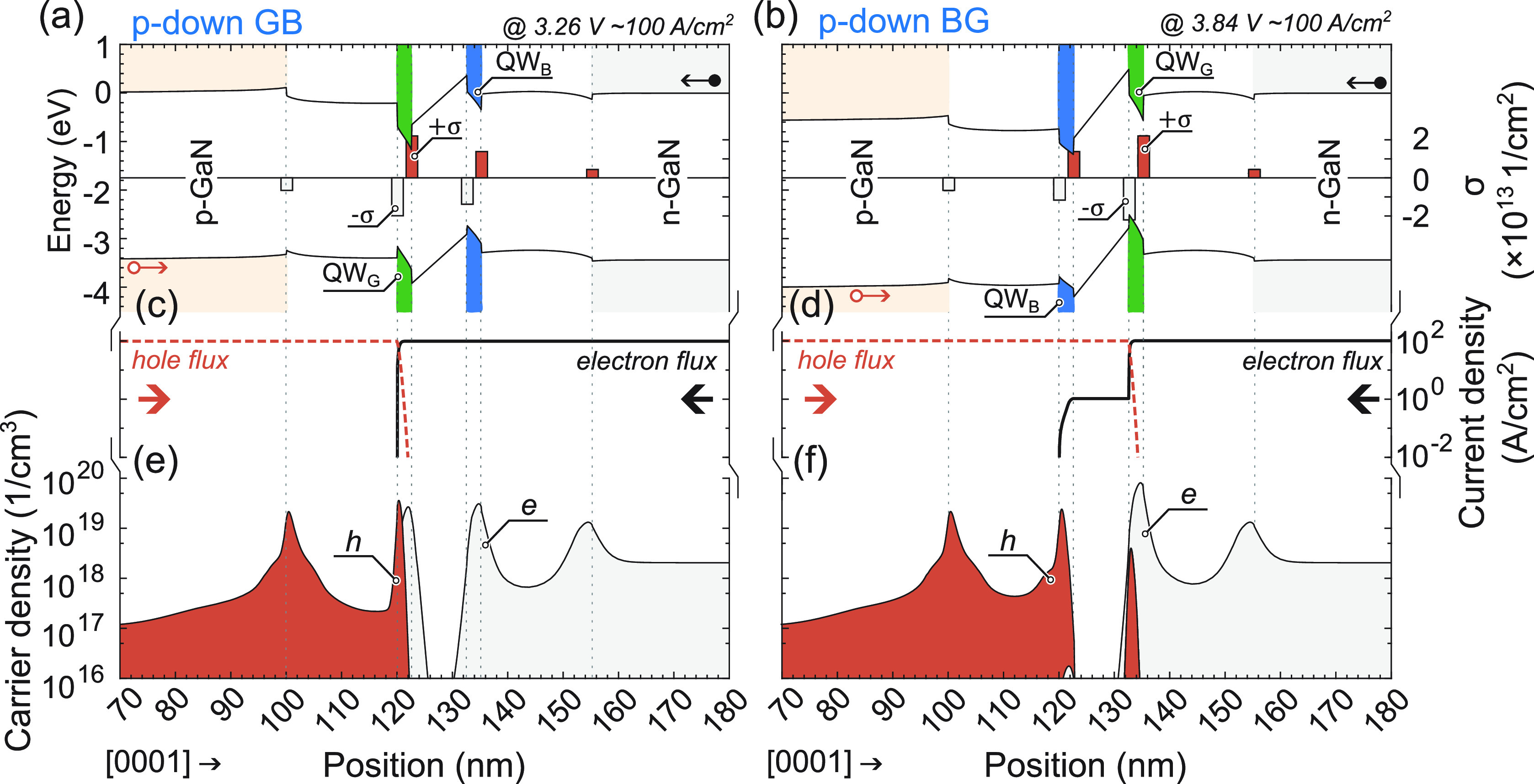
For “p-down” LEDs: (a,b) band structures at ∼100 A/cm^2^ with σ-polarization sheet charge at interfaces, (c,d) current density vs position, and (e,f) carrier density vs position. Left column describes p-down GB LED, right column describes p-down BG LED.

The results and distinctions between the two types of devices can be explained through device simulations, as illustrated in Figures 8 and 9 for “p-up” and “p-down” LEDs, respectively. The energy band diagrams, along with current density and carrier concentration plotted against stack position, were calculated using self-consistent Schrödinger-Poisson and drift-diffusion solver.^30^

In Figure 8, it is evident that for both BG and GB configurations of “p-up” LEDs, the majority of electron flux overflows the first QW on their path, whereas holes do not (2 orders of magnitude lower hole flux reaches the second QW). The magnitude of the electron overflow is influenced by the built-in electric field in the QW. Higher In content leads to increased σ in the QW, leading to higher acceleration of electrons in the QW promoting overflow. In this case, the hole mobility serves as the primary bottleneck, limiting recombination in QWs located farther away from the p-type. Consequently, in “p-up” LEDs, a greater concentration of holes and electrons occurs in the QW closest to the p-type, as depicted in Figure 8e,f. Only an increased bias results in holes spilling over more distant QWs. These two mechanisms explain the obtained relation between electroluminescence peaks for different current densities presented in Figure 5a,b. This case represents a conventional approach to LEDs, with similar effects widely discussed in the literature.^8−11^

In the case of “p-down” LEDs, where current flow direction is inversed with respect to previously described LEDs, the current flow and carrier distribution exhibit notable differences. In the sample with the GB QW configuration, as illustrated in Figure 9a,d,f, electrons overflow the blue QW, while holes are restricted to the green QW, which is the first in their path. As a result, recombination occurs exclusively in the green QW, which is consistent with the spectra presented in Figure 5c. Notably, only at high current densities, exceeding 1 kA/cm^2^, can holes reach the blue QW and recombine radiatively.

Unexpectedly, for the sample with the BG QW configuration presented in Figure 9b,c,e, the behavior is the opposite. There is an exceptionally low overflow of electrons through the green QW, which is the first in their path. The majority of electrons recombine in the green QW, leading to 2 orders of magnitude lower electron flux reaching the second, blue QW. From the perspective of holes, in the first QW in their path (blue QW), there is no possible recombination as the QW is devoid of electrons. Consequently, holes are forced to overflow from the blue QW to the next green QW, resulting in a predominant spectral contribution from the green QW, as depicted in Figure 5d. An increase in an external bias leads to electron spilling over the blue QW, and faint blue emission can be observed above 50 A/cm^2^.

The polarization sheet charge, which promotes carrier overflow in “p-up” LEDs, for “p-down” LEDs inhibit it. This effect is dependent on the magnitude of the polarization charge, resulting in stronger carrier flux blocking properties with an increasing In content of the QW. In “p-down” devices, this effect confines carriers within the QW with the highest In content. Furthermore, due to this inherent effect, “p-down” LEDs do not necessitate a dedicated electron-blocking layer, as each QW/barrier interface naturally restrains overflow and parasitic recombination originating caused by it.

## 4 Conclusions

The findings demonstrate how the inversion of the current flow direction with respect to the built-in field causes a change in carrier transport and distribution within double QW LEDs obtained on Ga-polar GaN. In the conventional “p-up” LED, the radiative recombination is limited by the low mobility of holes. In contrast, for “p-down” LEDs, carrier transport is limited by the potential barrier at the QW interface, confining carriers to the QW with the highest In content. Consequently, recombination from the QW farther from the p-type dominates the electroluminescence spectra, even though the mobility of holes remains low in comparison to electrons. The multi-QW structure results in a more pronounced asymmetry of carrier distribution than observed in “p-up” devices.

This highlights the effectiveness of the barrier at the QW interface as a blocking element for both holes and electrons. This gives valuable insights into how to design such devices, i.e., the “p-down” devices do not require a dedicated AlGaN electron blocking layer, and multiple QW structures do not yield the same benefits as seen in “p-up” LEDs. One potential solution to overcome this limitation might be employing a single QW design with increased thickness, exceeding 10 nm.^31−33^

Furthermore, the “p-down” construction mimics the orientation of electric fields as if it were grown on an N-polar substrate, while maintaining the high efficiency characteristic of Ga-polar grown LEDs. The results suggest that the design principles effective for “p-down” LEDs can be applied to N-polar LEDs as well.

## Supporting Information Available

EQE for multicolor emission, data reproducibility for seven devices, Energy at maximum electroluminescence intensity, current density at maximum EQE, and IV characteristics (PDF)
