# Supplementary material for: Harnessing III-Nitride Built-In Field in Multi-Quantum Well LEDs
Source: ACS Appl Mater Interfaces. 2024 Apr 26;16(18):24021–8. doi: 10.1021/acsami.4c02084 (PMC11082851; doi:10.1021/acsami.4c02084)
Supplement: Supplementary file 1 — am4c02084_si_001.pdf [file am4c02084_si_001.pdf]

# Supporting Information

# Harnessing III-Nitride Built-In Field in Multi-Quantum Well LEDs

Mikołaj Chlipała[1*], Henryk Turski[1,2]

**[*]Corresponding Author**

**Mikołaj Chlipała -** Institute of High Pressure Physics Polish Academy of Sciences, PAS, 01-142 Warsaw, Poland; Email: chlipala@unipress.waw.pl

**Henryk Turski -** Institute of High Pressure Physics Polish Academy of Sciences, PAS, 01-142 Warsaw, Poland; School of Electrical and Computer Engineering, Cornell University, 14853 Ithaca, NY, USA

## Experimental - external quantum efficiency (EQE)

Using measured optical power ($P$) external quantum efficiency (EQE) can be calculated as follows:

$$EQE_X = \frac{P/h\nu}{I_F/q} \ ,$$

where: $I_F$, forward current; $q$, elementary charge; $h$, Planck constant; $\nu$, frequency [1]. EQE also give as insight into the inner quality of QW following ABC model where:

$$EQE = \eta_e \eta_{inj} \frac{Bn^2}{An + Bn^2 + Cn^3},$$

where: $\eta_e$ is extraction efficiency, $\eta_{inj}$ is injection efficiency, $n$ is carrier concentration, $A$ is coefficient attributed to the Shockley—Read—Hall (SRH) nonradiative recombination, $B$ the radiative recombination coefficient and $C$ is Auger nonradiative coefficients.[2] As EQE depends on the photon energy, for double color LED we got the contribution of each QW to optical power based on spectra. We defined a total EQE ($EQE_{total}$) as:

$$EQE_{total} = EQE_B + EQE_G,$$

where subscripts "B" and "G" denotes contribution from blue and green QW.

## Results – Sample uniformity

Fig. 1 illustrates the shift in electroluminescence (EL) versus voltage for six individual devices, each measuring 100x100 µm². Energy data was derived by fitting Gaussian functions to EL spectra for the blue and green Quantum Wells (QW). While there is slight variation observed between devices, the overall trend remains consistent across all samples.

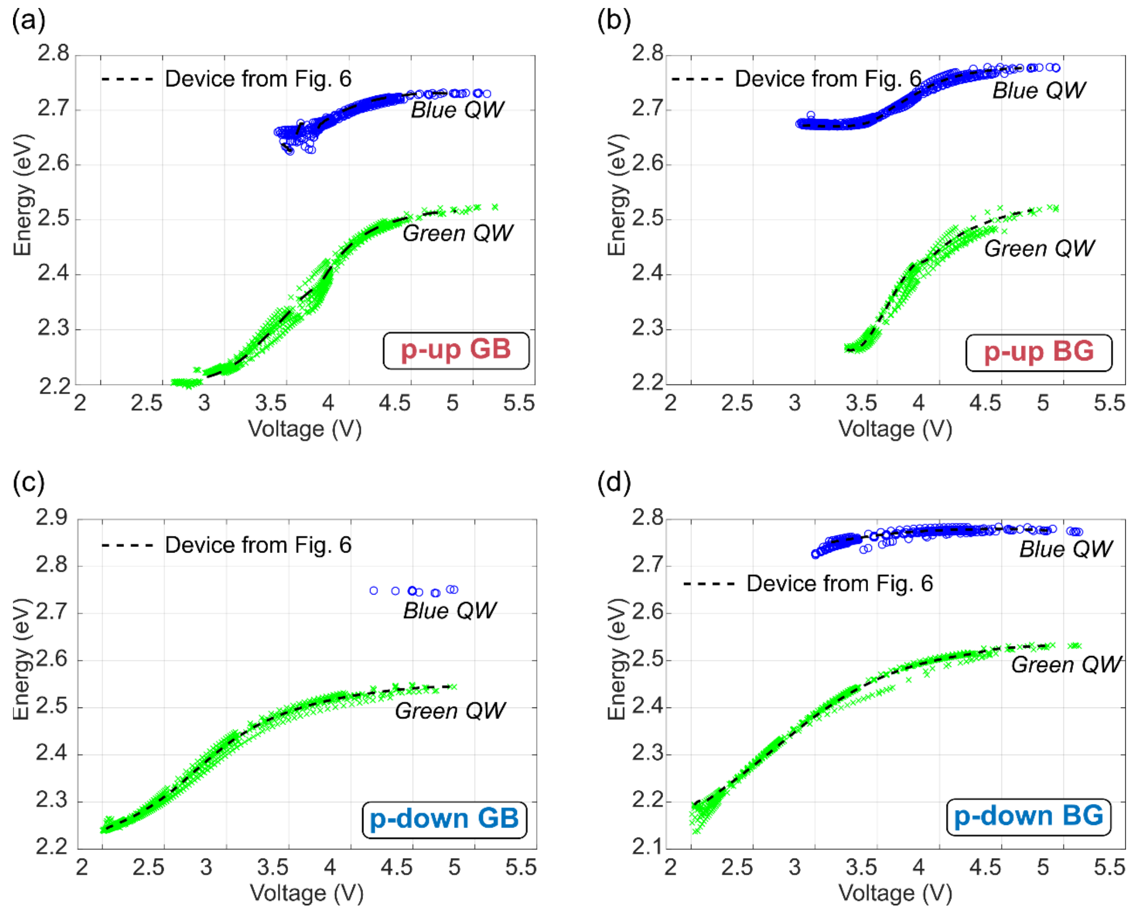


Figure 1 Energy at maximum electroluminescence intensity for blue and green QW.  Measured for 6 devices wit contact are 100x100 μm$^2$ for each sample.

Table 1 contains current density at maximum EQE for seven devices, each with an area of 100x100 μm², across four samples.. he device with characteristics closest to the average was selected for Fig. 7 in the main text. There is noticeable variation in the qualities of the devices. However, this variation does not impact the conclusions drawn in the study.

Table 1 Current density at maximum EQE for 7 devices for 4 samples.

| **Name** | **Current Density at maximum EQE (A/cm$^2$)** | | | | | | | |
|---|---|---|---|---|---|---|---|---|
| | **LED#1** | **LED#2** | **LED#3** | **LED#4** | **LED#5** | **LED#6** | **LED#7** | **Average** |
| p-down GB | 184 | 208 | 222 | 236 | 196 | 143 | 208 | **199** |
| p-down BG | 308 | 156 | 167 | 156 | 167 | 159 | 205 | **188** |
| p-up GB | 17 | 40 | 19 | 22 | 78 | 24 | 89 | **41** |
| p-up BG | 82 | 136 | 136 | 89 | 75 | 53 | 55 | **90** |

Figure 2 depicts the IV characteristics of seven devices, each with an area of 100x100 μm², across four samples. All devices exhibits uniform electrical characteristics.

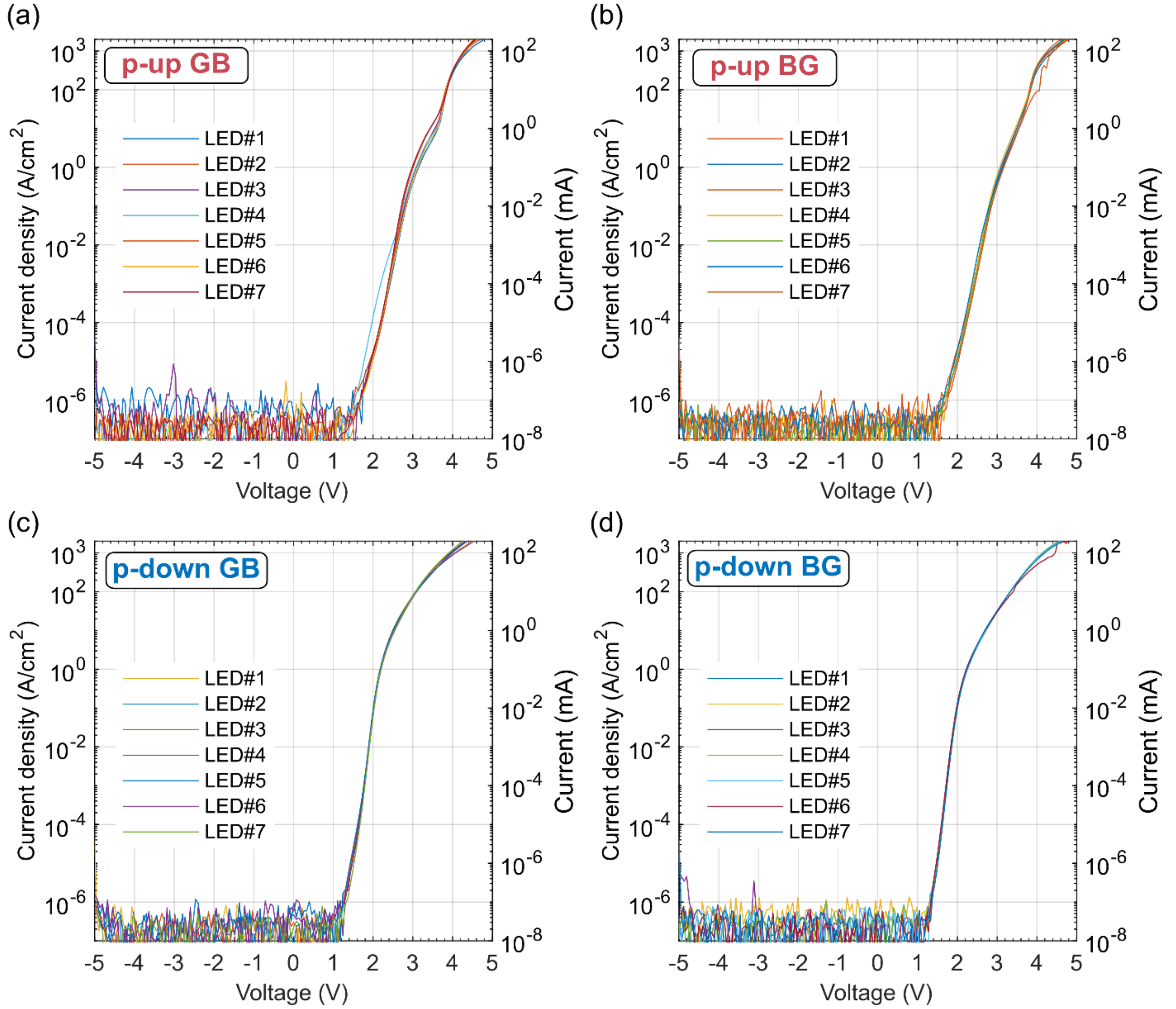


Figure 2 IV characteristics seven devices, each with an area of 100x100 μm², across four samples.

## References


(1) Shim, J. I.; Shin, D. S. Measuring the Internal Quantum Efficiency of Light-Emitting Diodes: Towards Accurate and Reliable Room-Temperature Characterization. *Nanophotonics* **2018**, 7 (10), 1601−1615.

(2) Dai, Q.; Schubert, M. F.; Kim, M. H.; Kim, J. K.; Schubert, E. F.; Koleske, D. D.; Crawford, M. H.; Lee, S. R.; Fischer, A. J.; Thaler, G.; Banas, M. A. Internal Quantum Efficiency and Nonradiative Recombination Coefficient of GaInN/GaN Multiple Quantum Wells with Different Dislocation Densities. *Appl. Phys. Lett.* **2009**, 94 (11), 111109.
